# Being Pregnant during COVID-19: Exploring the COVID-19 Related Beliefs, Behaviors, and Birth Outcome among Users of a Pregnancy App

**DOI:** 10.3390/ijerph21010034

**Published:** 2023-12-26

**Authors:** Hui Huang, Olivia Ceavers, Maria Pinzon Iregui, Melissa M. Howard

**Affiliations:** 1 School of Social Work, University of Texas at Arlington, Arlington, TX 76010, USA; 2 Department of Health Promotion & Disease Prevention, Robert Stempel College of Public Health & Social Work, Florida International University, Miami, FL 33199, USA; oceav001@fiu.edu (O.C.); mpinzoni@fiu.edu (M.P.I.); 3 Department of Health Promotion and Behavior, College of Public Health, University of Georgia, Athens, GA 30602, USA; melissa.howard@uga.edu

**Keywords:** pregnant people, COVID-19, vaccination, birth outcome, fetal movement tracking app

## Abstract

Being pregnant during COVID-19 increases the risk of experiencing severe COVID-19 symptoms, which in turn increases the risk of complications. This study aimed to examine COVID-19-related beliefs, behaviors, and birth outcomes among users of Count the Kicks (CTK), a fetal movement tracking app. This study used the End of Pregnancy Survey data from 1037 CTK users. We used descriptive analyses to analyze COVID-19-related beliefs and behaviors and used the chi-square statistic and Z statistic to examine factors associated with vaccination and birth outcome. Nearly half of the survey respondents reported not being concerned that in-person prenatal visits might lead to COVID-19 exposure. Most respondents (65.9%) had already received the COVID-19 vaccine at the time of the survey. The vaccination rate was statistically significantly lower among African Americans than the remaining race/ethnicity groups (mostly white). The healthy birth rate is lower among respondents with high-risk pregnancies, while the stillbirth rate is highest among Hispanics. Vaccination status was not associated with the likelihood of a healthy birth. Our findings confirmed that vaccination does not compromise birth outcomes, further contributing to the existing evidence of COVID vaccine safety during pregnancy. This study also demonstrates an example of using data from a pregnancy app to facilitate research on app users in real-time.

## 1. Introduction

The COVID-19 pandemic has had a profound impact on various areas of health for pregnant people, including prenatal care visits and physical and mental health outcomes. In March 2020, when the President of the United States (US) announced the virus was spreading in the US, the delivery of medical care transitioned to a combination of in-person visits and telehealth visits to reduce transmission among obstetric patients [1,2,3]. The urgent need to safely deliver prenatal care has resulted in an increase in the utilization of telemedicine services and mobile application technology worldwide [4,5,6,7]. Given the shift in prenatal care, some research has cited the pandemic as contributing to an adverse effect on the healthcare system and delivery of medical care, resulting in increased stillbirths and maternal deaths [8,9]. Several studies have also shown an increase in mental health problems, with higher depression and anxiety scores among pregnant people during home quarantine and increased pregnancy-related anxiety and stress levels [10,11]. As a result of decreased in-person prenatal visits and a loss of social support, web-based technology, such as mobile applications, has served as an alternative option to provide pregnant people with awareness and knowledge about their pregnancy [12,13,14]. Count the Kicks (CTK) is one of the pregnancy-related mobile applications that aim to increase awareness of fetal activity among pregnant people [15]. CTK provides a virtual platform for pregnant people to conduct a daily kick count throughout the third trimester. The benefit is that pregnant women who consistently monitor fetal movement are likely to identify abnormal fetal movement timely, which can lead them to seek medical attention and ultimately reduce the incidence of preventable stillbirths.

### 1.1. COVID Vaccination among Pregnant Women

On 11 August 2021, the CDC announced the COVID-19 vaccine is safe for pregnant people, and the benefits of receiving a vaccine outweigh any known or potential risks [16]. While recommendations for and knowledge of vaccination for pregnant people have changed throughout the pandemic, the American College of Obstetricians and Gynecologists (ACOG) and the Society for Maternal Fetal Medicine have continued to advocate for the vaccination of pregnant people [17]. The fact that pregnant people who contract COVID-19 are at increased risk for adverse pregnancy outcomes further emphasizes the importance of vaccinating people who are or may become pregnant [18,19]. Several studies analyzed acceptance and willingness to receive COVID-19 vaccinations [20,21,22,23]. However, limited data exist regarding the actual COVID-19 vaccination status during pregnancy. To our knowledge, there is no current published research that measures the percentage of people who received the COVID vaccine before, during, or after their pregnancy.

### 1.2. COVID Vaccination and Birth Outcome

Concerns about the safety of COVID-19 vaccines are a barrier to vaccine uptake among pregnant people, despite studies that confirm the safety of COVID-19 vaccination during pregnancy, with no association between COVID-19 vaccination and adverse birth outcomes, such as increased risk for stillbirth [24], preterm birth, or small for gestational age (SGA) at birth [25]. Additionally, research assessing vaccine safety and uptake has shown a trend of higher vaccine hesitancy among certain demographics, including pregnant people who are younger, non-White, and of lower socioeconomic status [26]. One study that used CDC vaccination data reported that only 16.3% of pregnant women had received one dose of the vaccine as of May 2021, with the lowest vaccination rates among Hispanic (11.9%) and non-Hispanic Black women (6.0%) [27].

This study explores beliefs, behaviors, and pregnancy-related outcomes (including changes in prenatal care, perceived anxiety levels, and birth outcomes) during the COVID-19 pandemic among a sample of people who used CTK, a pregnancy-related app. We also present empirical measurements of the percentages of pregnant people who received the COVID-19 vaccine prior to, during, or immediately after pregnancy and the reasons for receiving or not receiving the vaccination. This study aims to address three research questions:

(1) How did CTK-using pregnant people respond to the COVID-19 pandemic? For example, did they receive the COVID-19 vaccine? Did they experience changes to their prenatal visits?
(2) Which factors are associated with the COVID-19 vaccination status of CTK-using pregnant people?
(3) Which factors are associated with birth outcomes among CTK-using pregnant people?

## 2. Methods

### 2.1. Sample

This study used data obtained from a data-sharing agreement between the researchers and Healthy Birth Day, Inc., the developer of CTK. The study sample includes CTK users who answered the “End of Pregnancy Survey” administered through an app-based push notification between 16 July 2021 and 22 December 2021. This time window was selected as survey questions related to COVID-19 were implemented beginning July 2021. Only survey respondents from the US were included in the sample. Out of the 1319 survey respondents from the US, 282 (21%) with missing data on their birth story (i.e., self-reported birth outcome) were excluded from the sample due to the importance of this variable. The final sample included 1037 survey respondents from the US. In the sample, 5.7% are African American, 4.7% are Asian, 59.3% are Caucasian, 8.8% are Hispanic, 7.6% are other, and 13.9% did not report their race or ethnicity. This study was conducted in accordance with prevailing ethical principles and reviewed by an Institutional Review Board of the second and third authors’ universities.

### 2.2. Measure

The study measure is the End of Pregnancy Survey that CTK pushed to users 14 days after their self-reported due date. According to Healthy Birth Day, Inc., the overall response rate for the survey is 12% (10,886 out of 91,041 total users to date). The survey contains 18 questions. The first eight questions have been implemented since October 2018. The last 10 questions were added in July 2021 to assess pregnant women’s behaviors related to COVID-19.

### 2.3. Analytic Approaches

We used descriptive analyses, including mean, standard deviation, and percentage, to answer the first research question regarding COVID-19-related behaviors. We used a chi-square statistic and a Z statistic to answer the second and third research questions related to factors associated with vaccination status and birth outcome.

## 3. Results

### 3.1. COVID-19 and Vaccination-Related Factors

As shown in Table 1, when asked how concerned they are that visiting their prenatal provider (e.g., OB/GYN, nurse, or midwife) in person might lead to COVID-19 exposure, close to half (43.4%) of the sample reported not at all concerned. Nearly a third (32.4%) reported being slightly concerned. Only 14.6% reported being somewhat concerned, 7.3% reported being moderately concerned, and 2.3% reported being extremely concerned.

Only 12% (N = 127) of the sample reported their provider replaced some of their in-person prenatal visits with telehealth visits (via phone or computer). Even among those for whom in-person prenatal visits were replaced with telehealth visits, most (65%) experienced only a slight reduction, indicating one or two (1–2) prenatal visits were replaced with telehealth.

At the time of the survey, a majority (65.9%) of the sample had already received the COVID-19 vaccine. Ten percent of the sample (10.1%) reported having received it before pregnancy, 51.7% reported having received it during pregnancy, and 4.1% reported having received it after pregnancy. Only 34.1% of the sample reported not receiving a COVID-19 vaccine at all at the time of survey completion.

When those who received the COVID-19 vaccine were asked about the top reasons why they got vaccinated, the three commonly mentioned reasons were to pass the benefits of vaccination on to their baby, keep themselves safe, and keep their families safe. Those who did not get vaccinated also reported their reasons not to receive the vaccine. The three commonly mentioned reasons were concern that the vaccine would harm the fetus/baby, lack of trust in the safety of COVID-19 vaccines, and concern about side effects from the vaccine.

### 3.2. Factors Associated with Vaccination

As shown in Table 2, we examined the association between high-risk pregnancy and vaccination status, both of which were self-reported in the End of Pregnancy Survey. When vaccination status is treated as a categorical variable with four categories, there is no statistically significant association between the status of a high-risk pregnancy and vaccination status. When vaccination status was treated as a binary variable (vaccinated or not), the result was similar. There was no statistically significant association between the status of a high-risk pregnancy and vaccination status. We also examined the association between race and vaccination status. When vaccination status was treated as a categorical variable with four categories, there was a statistically significant association between race/ethnicity and vaccination status. When vaccination status was treated as a binary variable, the result was similar. The vaccination rate was statistically significantly lower among the respondents who identified themselves as African American or other as compared with the remaining race/ethnicity groups (Asian, Caucasian, Hispanic, or missing).

### 3.3. Factors Associated with Birth Outcome

As shown in Table 2, we also examined the association between the status of high-risk pregnancy and birth outcome, both of which were self-reported in the End of Pregnancy Survey. When birth outcome was treated as a categorical variable with five categories, there was a statistically significant association between the status of a high-risk pregnancy and vaccination status. Respondents with high-risk pregnancies were less likely to report that their baby was born healthy within two weeks of the due date. When birth outcome was treated as a categorical variable with four categories, the result was similar.

We also examined the association between race and birth outcomes. When birth outcome was treated as a categorical variable with five categories, there was a statistically significant association between race and birth outcome. We also calculated Z statistics to analyze the intergroup differences. The results from Z statistics showed that the stillbirth rate was statistically significantly higher among the respondents who identified themselves as Hispanic as compared with the other race groups. When birth outcome was treated as a categorical variable with four categories, the result was similar.

Lastly, we examined the association between vaccination status and birth outcome. When birth outcome was treated as a categorical variable with five categories, there was no statistically significant association between vaccination status and birth outcome. In other words, vaccination did not compromise birth outcomes. When birth outcome was treated as a categorical variable with four categories, there was still no statistically significant association.

## 4. Discussion

This research explored the COVID-19-related beliefs, behaviors, and pregnancy-related outcomes among people who used CTK during the COVID-19 pandemic.

### 4.1. Vaccination during the Perinatal Period

A large body of evidence suggests increased health risks for pregnant people who contract COVID-19 and supports the safety of vaccination during pregnancy. In our study, most participants received the COVID vaccine in the perinatal period (65.9%); more than half received it during pregnancy (51.8%). The self-reported vaccination rate in our study was close to the rate of intention to vaccinate reported in one study (52.7%), while it was higher than the rate of intention to vaccinate reported in another study (41%) [20,23]. A key finding in our study is that vaccination does not compromise birth outcomes, further contributing to the existing evidence of COVID vaccine safety during pregnancy.

### 4.2. Prenatal Care and COVID-19 Exposure Concerns

Previous studies have reported a shift in prenatal care at the start of the COVID-19 pandemic in the United States. Jamieson and Rasmussen, for example, discussed potential adverse effects on the delivery of obstetric care, which could result in increased stillbirth and maternal deaths, with the primary concerns of increased risk for severity of disease, ICU admission, and ventilation [9]. In our study, participants did not report experiencing a large shift in how they received prenatal care. Rather, most obstetric visits remained in-person and were seldom replaced by telehealth.

The participants in this study were asked how concerned they were that visiting their prenatal provider (e.g., OB/GYN, nurse, or midwife) in person might lead to COVID-19 exposure, and close to half of the sample reported not being at all concerned. There was a small group of people who reported being moderately concerned (7.3%) and extremely concerned (2.3%). This finding differs from other studies that reported much higher levels of exposure concern and anxiety among pregnant people [11]. It is important to note that the present study took place at a time when vaccines had been approved and recommended during pregnancy, no lockdown orders were in place, and OB/GYN offices were returning to their pre-pandemic care delivery models. This might offer a further explanation of this difference in findings from our study as compared to other studies conducted earlier during the pandemic.

### 4.3. Acceptance and Refusal of the COVID Vaccine

Reasons why our study participants decided to or not to get the vaccine are consistent with other studies that measured attitudes towards acceptance of the COVID-19 vaccination. Their reasons are to protect the safety of their baby, themselves, and their families. Wanting to protect their baby is the most common reason for both acceptance and refusal of the vaccine, which is consistent with the findings from other published work [20]. A previous review of the literature also reported that reasons for refusal of the COVID-19 vaccination are related to concerns about its safety, such as perceiving a vaccine produced in a rash as too dangerous [28]. Therefore, it is important to increase public awareness of the safety of COVID-19 vaccines. Strategies to increase this kind of public awareness should include engaging a broad network of trusted spokespersons to disseminate vaccine-affirming messages and making COVID-19 vaccines available at familiar and convenient locations that feel safe to the public [29].

### 4.4. Race/Ethnicity, Vaccination Status, and Birth Outcomes

Our findings of an association between race/ethnicity and vaccination status are consistent with prior studies. Collecting data from the UK, Blakeway et al. reported lower vaccine uptake among people who report non-White ethnicity [26]. Additionally, there have been differences observed in attitudes towards vaccination among racial groups, with a higher percentage of Black pregnant women reporting they would not follow their OB/GYN’s recommendation to get the COVID vaccine [23]. We observed similar results in our participant population, with the lowest vaccine uptake among people who reported being Black/African American, or other as their race. A previous study on COVID vaccine hesitancy among African Americans reported their vaccine hesitancy is related to historical mistrust of government and pharmaceutical companies conducting unethical healthcare research among Black populations, uncertainty on the effect of the vaccine on people with pre-existing health conditions such as diabetes, social media misinformation, and political affiliation (higher vaccine hesitancy among Republican political affiliation) [30].

There was an unexpected finding when examining the association between race and birth outcomes. The self-reported stillbirth rate was found to be highest among Hispanics compared to the other race/ethnicity groups, which is inconsistent with literature that reported the highest rates of preterm birth and stillbirth among Black/African Americans [31,32]. A possible explanation can be attributed to the small numbers of African Americans and Hispanics in our study sample, which limited the generalizability of our findings on African Americans and Hispanics.

### 4.5. Limitations

There are several limitations in our study, primarily due to the small sample size. First, the sample may not be fully representative of the broader population of pregnant people due to the nature of this study. Namely, it is conducted among the CTK users. It is likely that we have captured only the group of pregnant people who have access to and are more willing to use technology during pregnancy. Another limitation is the small number of participants who fully completed the End of Pregnancy Survey. Only 12% of the CTK users participated in the End of Pregnancy Survey.

## 5. Conclusions

Health-related pregnancy apps have the potential to become valuable tools in promoting a healthy pregnancy and may serve as useful support for prenatal care providers. These apps can also facilitate research on app users in real-time. As demonstrated in this study, data obtained through CTK were useful in assessing COVID-19-related beliefs, behaviors, and pregnancy-related outcomes among their users. This study showed that most app users who responded to the End of Pregnancy Survey got COVID-19 vaccines before or during their pregnancy. Moreover, getting vaccinated was not associated with a negative birth outcome, and most respondents experienced a healthy birth outcome.

## Figures and Tables

**Table 1:** Descriptive Analyses of COVID-19 Related Beliefs and Behaviors.

	N	Percent
During this pandemic, how concerned are you that visiting your prenatal provider (e.g., OB/GYN, nurse, or midwife) in person might lead to COVID-19 exposure?		
Not at all concerned	450	43.4
Slightly concerned	336	32.4
Somewhat concerned	151	14.6
Moderately concerned	76	7.3
Extremely concerned	24	2.3
Did your medical provider replace some of your in-person prenatal visits with telehealth visits (via phone or computer)?		
No	910	87.8
Yes	127	12.2
[Of “Yes” responses immediately above] How much did your medical provider (e.g., OB/GYN, nurse, or midwife) reduce your in-person prenatal visits due to COVID-19 precautions?		
Slight reduction—Only one or two (1–2) of my prenatal visits were replaced with telehealth	83	8.0
Some reduction—Three or four (3–4) of my prenatal visits were replaced with telehealth	30	2.9
Moderate reduction—Five or six (5–6) of my prenatal visits were replaced with telehealth	10	1.0
Extreme reduction—Seven or more (7+) of my prenatal visits were replaced with telehealth	4	0.4
Did you receive a COVID-19 vaccine?		
Yes, I received it before my pregnancy	105	10.1
Yes, I received it during my pregnancy	536	51.7
Yes, I received it after my pregnancy	43	4.1
No, I have not received a COVID-19 vaccine	353	34.0
[Of “Yes” to vaccine respondents] What is your PRIMARY reason to get the COVID-19 vaccine? (Choose one)		
I wanted to pass the benefits of vaccination on to my baby	281	27.1
I wanted to keep myself safe	153	14.8
I wanted to keep my family safe	149	14.4
I wanted to keep my community safe	14	1.4
I wanted to feel safe around other people	20	1.9
I have a chronic health problem, like asthma or diabetes	10	1.0
My doctor told me to get a COVID-19 vaccine	11	1.1
I believe life will not go back to normal until most people get the COVID-19 vaccine	40	3.9
Other	7	0.7
[Of “Yes” to vaccine respondents] What is your SECONDARY reason to get the COVID-19 vaccine? (Choose one)		
I wanted to pass the benefits of vaccination on to my baby	180	17.4
I wanted to keep myself safe	201	19.4
I wanted to keep my family safe	138	13.3
I wanted to keep my community safe	33	3.2
I wanted to feel safe around other people	64	6.2
I have a chronic health problem, like asthma or diabetes	4	0.4
My doctor told me to get a COVID-19 vaccine	16	1.5
I believe life will not go back to normal until most people get the COVID-19 vaccine	44	4.2
Other	5	0.5
[Of “Yes” to vaccine respondents] What is your THIRD reason to get the COVID-19 vaccine? (Choose one)		
I wanted to pass the benefits of vaccination on to my baby	89	8.6
I wanted to keep myself safe	107	10.3
I wanted to keep my family safe	171	16.5
I wanted to keep my community safe	82	7.9
I wanted to feel safe around other people	90	8.7
I have a chronic health problem, like asthma or diabetes	9	0.9
My doctor told me to get a COVID-19 vaccine	37	3.6
I believe life will not go back to normal until most people get the COVID-19 vaccine	91	8.8
Other	9	0.9
[Of “No” to vaccine respondents] What is your PRIMARY reason for NOT receiving the COVID-19 vaccine? (Choose one)		
During my pregnancy, I was concerned that the vaccine would harm the fetus/baby	167	16.1
I am concerned that the vaccine will affect fertility	15	1.4
I am concerned that the vaccine will affect breastfeeding	10	1.0
I am allergic to vaccines	2	0.2
I am concerned about the side effects of the vaccine	27	2.6
I do not think COVID-19 vaccines work very well	9	0.9
I do not trust the safety of COVID-19 vaccines	66	6.4
I do not believe the COVID-19 pandemic is as bad as some people say it is	5	0.5
I have been too busy/I have not gotten around to it	11	1.1
Other	41	4.0
[Of “No” to vaccine respondents] What is your SECONDARY reason NOT to receive the COVID-19 vaccine? (Choose one)		
During my pregnancy, I was concerned that the vaccine would harm the fetus/baby	75	7.2
I am concerned that the vaccine will affect fertility	50	4.8
I am concerned that the vaccine will affect breastfeeding	33	3.2
I am allergic to vaccines	2	0.2
I am concerned about the side effects of the vaccine	70	6.8
I do not think COVID-19 vaccines work very well	22	2.1
I do not trust the safety of COVID-19 vaccines	40	3.9
I do not believe the COVID-19 pandemic is as bad as some people say it is	9	0.9
I have been too busy/I have not gotten around to it	16	1.5
Other	35	3.4
[Of “No” to vaccine respondents] What is your Third reason for NOT receiving the COVID-19 vaccine? (Choose one)		
During my pregnancy, I was concerned that the vaccine would harm the fetus/baby	52	5.0
I am concerned that the vaccine will affect fertility	34	3.3
I am concerned that the vaccine will affect breastfeeding	44	4.2
I am allergic to vaccines	1	0.1
I am concerned about the side effects of the vaccine	77	7.4
I do not think COVID-19 vaccines work very well	17	1.6
I do not trust the safety of COVID-19 vaccines	48	4.6
I do not believe the COVID-19 pandemic is as bad as some people say it is	6	0.6
I have been too busy/I have not gotten around to it	20	1.9
Other	53	5.1

**Table 2:** Binary Analysis of Factors Related to Vaccination and Birth Outcomes.

Did You Receive a COVID-19 Vaccine?									Chi-Square	*p*
high-risk pregnancy	Yes, I received it before my pregnancy		Yes, I received it during my pregnancy		Yes, I received it after my pregnancy		No, I have not received a COVID-19 vaccine			
No	70 (10.5%)		330 (49.6%)		28 (4.2%)		237 (35.6%)		6.183	0.403
Yes	31 (9.0%)		193 (56.1%)		15 (4.4%)		105 (30.5%)			
I do not know	4 (14.3%)		13 (46.4%)		0 (0.0%)		11 (39.3%)			
high-risk pregnancy	Yes, I received a COVID-19 vaccine		No, I have not received a COVID-19 vaccine							
No	428 (64.4%)		237 (35.6%)						2.995	0.224
Yes	239 (69.5%)		105 (30.5%)							
I do not know	17 (60.7%)		11 (39.3%)							
Race/ethnicity	Yes, I received it before my pregnancy		Yes, I received it during my pregnancy		Yes, I received it after my pregnancy		No, I have not received a COVID-19 vaccine			
Asian	8 (16.3%)		27 (55.1%)		1 (2.0%)		13 (26.5%)		56.289	<0.001
Black or African American	2 (3.4%)		26 (44.1%)		2 (3.4%)		29 (49.2%)			
White	51 (8.3%)		339 (55.1%)		24 (3.9%)		201 (32.7%)			
Hispanics	6 (6.6%)		54 (59.3%)		3 (3.3%)		28 (30.8%)			
Other	4 (5.1%)		33 (41.8%)		5 (6.3%)		37 (46.8%)			
Missing	34 (23.6%)		57 (39.6%)		8 (5.6%)		45 (31.3%)			
Race/ethnicity	Yes, I received a COVID-19 vaccine		No, I have not received a COVID-19 vaccine							
Asian	36 (73.5%)		13 (26.5%)						14.430	0.013
Black or African American	30 (50.8%)		29 (49.2%)							
White	414 (67.3%)		201 (32.7%)							
Hispanics	63 (69.2%)		28 (30.8%)							
Other	42 (53.2%)		37 (46.8%)							
Missing	99 (68.8%)		45 (31.3%)							
	**Please Tell Us Your Birth Story.**								**Chi-Square**	***p***
high-risk pregnancy	My baby was born healthy within two weeks of the due date	My baby was born premature but is now home		My baby was born premature and is still in the NICU		My baby died shortly after birth		My baby was born still		
No	629 (94.6%)	27 (4.1%)		6 (0.9%)		0 (0.0%)		3 (0.5%)	98.305	<0.001
Yes	261 (75.9%)	58 (16.9%)		20 (5.8%)		3 (0.9%)		2 (0.6%)		
I do not know	22 (78.6%)	3 (10.7%)		1 (3.6%)		0 (0.0%)		2 (7.1%)		
high-risk pregnancy	My baby was born healthy within two weeks of the due date	My baby was born premature		My baby died shortly after birth		My baby was born still				
No	629 (94.6%)	33 (5.0%)		0 (0.0%)		3 (0.5%)			97.652	<0.001
Yes	261 (75.9%)	78 (22.7%)		3 (0.9%)		2 (0.6%)				
I do not know	22 (78.6%)	4 (14.3%)		0 (0.0%)		2 (7.1%)				
Race/ethnicity	My baby was born healthy within two weeks of the due date	My baby was born premature but is now home		My baby was born premature and is still in the NICU		My baby died shortly after birth		My baby was born still		
Asian	41 (83.7%)	5 (10.2%)		3 (6.1%)		0 (0.0%)		0 (0.0%)	60.502	<0.001
Black or African American	58 (98.3%)	0 (0.0%)		1 (1.7%)		0 (0.0%)		0 (0.0%)		
White	544 (88.5%)	50 (8.1%)		19 (3.1%)		1 (0.2%)		1 (0.2%)		
Hispanics	79 (86.8%)	5 (5.5%)		2 (2.2%)		0 (0.0%)		5 (5.5%)		
Other	68 (86.1%)	9 (11.4%)		1 (1.3%)		0 (0.0%)		1 (1.3%)		
Missing	122 (84.7%)	19 (13.2%)		1 (0.7%)		2 (1.4%)		0 (0.0%)		
Race/ethnicity	My baby was born healthy within two weeks of the due date	My baby was born premature		My baby died shortly after birth		My baby was born still				
Asian	41 (83.7%)	8 (16.3%)		0 (0.0%)		0 (0.0%)			52.090	<0.001
Black or African American	58 (98.3%)	1 (1.7%)		0 (0.0%)		0 (0.0%)				
White	544 (88.5%)	69 (11.2%)		1 (0.2%)		1 (0.2%)				
Hispanics	79 (86.8%)	7 (7.7%)		0 (0.0%)		5 (5.5%)				
Other	68 (86.1%)	10 (12.7%)		0 (0.0%)		1 (1.3%)				
Missing	122 (84.7%)	20 (13.9%)		2 (1.4%)		0 (0.0%)				
Vaccination status	My baby was born healthy within two weeks of the due date	My baby was born premature but is now home		My baby was born premature and is still in the NICU		My baby died shortly after birth		My baby was born still		
No	316 (89.5%)	27 (7.6%)		6 (1.7%)		1 (0.3%)		3 (0.8%)	2.515	0.642
Yes	596 (87.1%)	61 (8.9%)		21 (3.1%)		2 (0.3%)		4 (0.6%)		
Vaccination status	My baby was born healthy within two weeks of the due date	My baby was born premature		My baby died shortly after birth		My baby was born still				
No	316 (89.5%)	33 (9.3%)		1 (0.3%)		3 (0.8%)			1.857	0.603
Yes	596 (87.1%)	82 (12.0%)		2 (0.3%)		4 (0.6%)				

## Data Availability

Data are unavailable since the study data are owned by the developer of the Count The Kicks app.
